# Molecular Prevalence and Genotyping of *Toxoplasma gondii* in Sheep Tissues Intended for Human Consumption in Shanxi Province, North China

**DOI:** 10.3390/ani15121685

**Published:** 2025-06-06

**Authors:** Xin-Sheng Lu, Jing Li, Chen Wang, Lu Wang, Xiao-Jing Wu, Xi-Long Yi, Ze-Xuan Wu, Wen-Bin Zheng, Xing-Quan Zhu

**Affiliations:** 1 Laboratory of Parasitic Diseases, College of Veterinary Medicine, Shanxi Agricultural University, Jinzhong 030801, China; luxinsheng2024@126.com (X.-S.L.); lijing1127x@163.com (J.L.); wangchen509260@163.com (C.W.); llyhtw1105@163.com (L.W.); wuxiaojing2017@163.com (X.-J.W.); wuzexuan0602@163.com (Z.-X.W.); 2 Research Center for Parasites & Vectors, College of Veterinary Medicine, Hunan Agricultural University, Changsha 410128, China; yxilong@126.com

**Keywords:** *Toxoplasma gondii*, sheep, prevalence, genotype, Shanxi Province, China

## Abstract

*Toxoplasma gondii* is one of the most widely distributed intracellular parasites worldwide, which can infect humans and a wide range of warm-blooded animals including sheep, with felines serving as its definitive host. *T. gondii* infection in sheep can lead to premature births, abortions and stillbirths, causing significant economic losses to the sheep industry. Sheep farming has become a key pillar of the agricultural economy in Shanxi Province, North China, but little is known about *T. gondii* infection in sheep in this province. In the present study, a total of 755 sheep tissue samples (682 muscle tissue samples and 73 lymphatic tissue samples) were collected from different markets in 10 different cities of Shanxi Province. The genomic DNA of all samples was extracted and the B1 gene of *T. gondii* was amplified by PCR. The B1 gene-positive samples were genotyped at 12 genetic markers employing the multilocus nested PCR-restriction fragment length polymorphism (Mn-PCR-RFLP). The molecular prevalence of *T. gondii* infection in sheep tissues in Shanxi Province was 20.5% (155/755). The *T. gondii* genotype ToxoDB#9 was identified in one positive *T. gondii* sample, with complete genotyping at all 12 genetic markers based on Mn-PCR-RFLP. This is the first report of molecular prevalence and genotype of *T. gondii* infection in sheep in Shanxi Province. These results reveal the widespread distribution of *T. gondii* in sheep in Shanxi, which is of significant public health importance.

## Simple Summary

*Toxoplasma gondii*, a widespread protozoan parasite responsible for significant health issues in humans and animals, poses economic risks to the global sheep industry. In Shanxi Province, North China, where sheep farming plays a vital role in local agriculture, little was known about the prevalence of *T. gondii* infection in the region’s sheep population. This study aimed to fill this knowledge gap by examining 755 sheep tissue samples (muscle and lymphatic tissues) collected from markets across 10 cities in Shanxi Province. *T. gondii* DNA was detected in 20.5% (155/755) of sheep samples using PCR. In addition, one strain was fully genotyped as ToxoDB#9 by Mn-PCR-RFLP, representing the first identification of this genotype in sheep in Shanxi Province. These findings highlight a notable prevalence of *T. gondii* in the region and provide critical insights into its genetic diversity, offering essential data for the development of targeted control strategies to mitigate infections in sheep, thereby safeguarding both human and animal health, and economic productivity in Shanxi’s agricultural sector.

## 1. Introduction

*Toxoplasma gondii* is a protozoan infecting approximately one-third of the human population worldwide, causing toxoplasmosis, one of the most common foodborne diseases [1,2]. This parasite can also infect almost all warm-blooded animals, primarily through the consumption of food or water contaminated with oocysts, bradyzoites or tachyzoites [2,3,4]. Although *T. gondii* infection is usually asymptomatic in immunocompetent people, it can cause serious complications in immunocompromised individuals [5]. In addition, infection during pregnancy can lead to miscarriage, stillbirth, or serious congenital defects, including blindness, intellectual disability and hydrocephalus [6].

In China, the national average *T. gondii* seroprevalence in humans is reported to be 8.2% [7], with significant regional variations. Some areas, particularly southwestern provinces, exhibit higher rates, reaching up to 20%. This discrepancy is attributed to differences in dietary habits, climate and diagnostic methods. While the overall seroprevalence of *T. gondii* is relatively low in Chinese population, the total number of infected individuals is substantial due to the country’s total population of 1.4 billion people [8]. Furthermore, a high proportion of workforce in China is involved in animal slaughtering, rearing and meat processing [9], and they face an elevated risk of *T. gondii* infection, primarily through accidental ingestion of tissue cysts from contaminated raw meat or exposure to oocyst-contaminated environments. Among livestock, sheep are susceptible intermediate hosts of *T. gondii* [10], and *T. gondii* infection in sheep can cause reproductive disorders such as stillbirths and abortions [11], significantly reducing the economic viability of sheep farming [12]. Furthermore, tissues and bodily fluids from acutely infected sheep, including mutton, offal, blood, exudates and milk, can serve as sources of *T. gondii* transmission to humans and other animals [13].

In fact, mutton is considered a good source of nutrition for human consumption because it is rich in protein, fatty acids, vitamins and minerals [14]. According to the latest data from the statistics bureau of China’s Shanxi Province, the number of sheep in Shanxi Province reached 7.17 million (https://tjj.shanxi.gov.cn/tjsj/tjnj/nj2023/zk/indexch.htm, accessed on 21 July 2024). The consumption of raw lamb or mutton containing *T. gondii* tissue cysts may be a potential source for human infection [15]. *T. gondii* tissue cysts can survive in cooked meat at an internal cooking temperature as low as 62.8 °C [16]. Thus, toxoplasmosis poses a significant threat to both public health and agricultural productivity, and its impact should not be underestimated. However, to date, investigation into the prevalence of *T. gondii* in sheep in Shanxi Province remains limited. A previous study focused solely on the seroprevalence of *T. gondii* in sheep in Shanxi Province, without identifying the genotype of the parasite [17]. These preliminary results were insufficient for a comprehensive evaluation of *T. gondii* infection in sheep in Shanxi Province.

Thus, this study aims to assess the molecular prevalence of *T. gondii* in muscle and lymphoid tissues of sheep collected from markets in northern, central and southern Shanxi by PCR targeting the *T. gondii* B1 gene [18,19], and to identify the genotypes of *T. gondii* in sheep using the multilocus nested PCR-restriction fragment length polymorphism (Mn-PCR-RFLP) [19,20]. The identification of molecular prevalence and genotype of *T. gondii* infection in sheep in Shanxi Province provides a scientific basis for the prevention and control of toxoplasmosis in sheep.

## 2. Materials and Methods

### 2.1. Sample Collection

**Figure 1:**
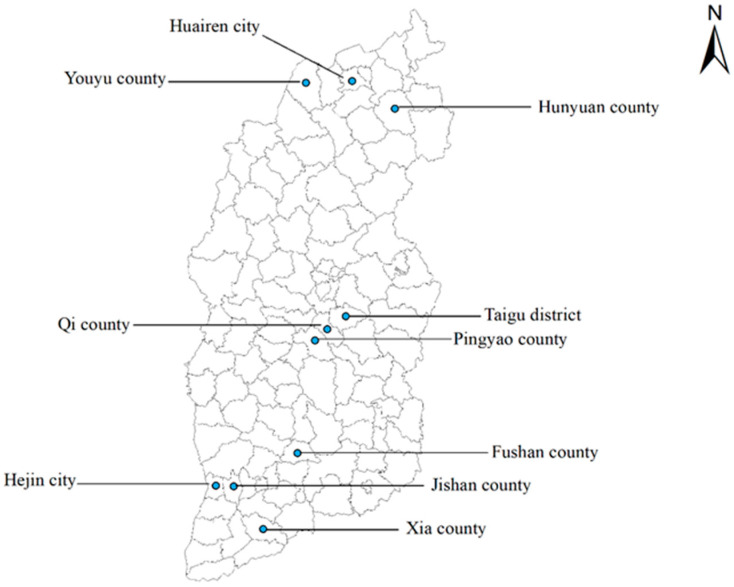
Sampling sites for commercially available sheep tissues in Shanxi Province, North China. The map was generated by ArcGIS 10.8 using data from the China National Geographic Information Public Service Platform.

Shanxi Province, located between latitudes 34°36′ and 40°44′ N and longitudes 110°15′ and 114°32′ E, covers 1.6% of China’s total land area, which has a temperate continental monsoon climate with an average annual temperature of 3~14 °C. From September to October 2023, a total of 755 sheep samples were collected or purchased from markets in 10 randomly selected cities spanning northern, central, and southern Shanxi (Figure 1), including 682 muscle samples (inner ridge, neck meat, front leg, and rear leg) and 73 lymphatic tissue samples (submandibular lymph nodes), with one tissue type being collected per sheep to ensure biological independence. Each sample was placed in a separate sterile plastic bag to prevent contamination, and relevant information such as sampling time, location and sample number were recorded. The collected samples were then transported to the Laboratory of Parasitic Diseases, College of Veterinary Medicine, Shanxi Agricultural University, and were stored in a −20 °C refrigerator until genomic DNA extraction.

### 2.2. DNA Extraction and PCR Amplification

For DNA extraction, 200 mg of each sample was homogenized using sterile scissors, followed by sequential treatment with 200 μL sodium dodecyl sulfate (SDS) and 20 μL proteinase K. The mixture was digested overnight in a constant-temperature water bath at 56 °C. DNA was then extracted from tissues using the TIANamp Genomic DNA Kit (TIANGEN, Beijing, China) according to the manufacturer’s instructions [18]. To detect *T. gondii*, semi-nested PCR was used to amplify the B1 gene of *T. gondii* according to previous descriptions [19,20], which has 35 copies in the *T. gondii* genome and is highly specific for *T. gondii* detection. The primers for the first round of amplification were F1 (5′-GGAACTGCATCCGTTCATGAG-3′) and R1 (5′-TCTTTAAAGCGTTCGTGGGTC-3′), and the cycling conditions were as follows: initial denaturation at 94 °C for 5 min, followed by 30 cycles of denaturation at 94 °C for 10 s, annealing at 57 °C for 10 s, extension at 72 °C for 30 s, and with a final extension at 72 °C for 5 min. For the second round of amplification, the primers F2 (5′-TGCATAGGTTGCAGTCACTG-3′) and R1 were used with similar cycling conditions, but with annealing at 62.5 °C for 10 s and extension at 72 °C for 15 s. The PCR reaction mixture (25 μL) consisted of 2.5 μL of 10 × PCR Buffer (Mg^2+^ free), 2 μL of MgCl_2_, 0.5 μL of dNTP, 0.2 μL of r-*Taq* polymerase (5 U/μL) (TAKARA, Dalian, China), and 2 μL of genomic DNA (as a DNA template for the primary PCR) or the primary PCR product (as DNA template for secondary PCR). After amplification, the secondary PCR products were examined by 2% agarose gel electrophoresis. The positive control (*T. gondii* DNA extracted from RH tachyzoites in cultured cells) and negative control (nuclease-free water) were included for each PCR.

### 2.3. Genotype Identification

To identify the genotypes of *T. gondii* in sheep, Mn-PCR-RFLP was used to amplify 12 genetic markers, including SAG1, SAG2 (5′SAG2, 3′SAG2 and altera. SAG2), SAG3, BTUB, GRA6, c22-8, c29-2, L358, PK1 and Apico [20,21]. In 2010, Su et al. developed a multi-locus PCR-RFLP approach targeting 12 genetic loci for genotyping *T. gondii*, a high-resolution and user-friendly technique that has been widely applied in *T. gondii* genotyping studies [21]. The PCR amplification was conducted in a 25 μL reaction volume containing 10 × PCR buffer, 0.2 μM of each primer, 200 μM dNTP, 2 mM MgCl_2_ and 0.2 U HotStart Taq DNA polymerase (TAKARA, Dalian, China). The amplification products of each genetic marker were digested with the corresponding restriction enzymes [21]. The genotyping results were compared to those of eight reference strains (GT1, PTG, CTG, MAS, TgCgCa1, TgCatBr5, TgCatBr64 and TgRsCr1) by analyzing the electrophoresis patterns. Subsequently, typing data were further analyzed using the ToxoDB database (http://toxodb.org/toxo/, accessed on 2 January 2025).

### 2.4. Statistical Analysis

In this study, SPSS version 20.0 (IBM, Chicago, IL, USA) was used to statistically analyze *T. gondii* prevalence across different regions via the Chi-square test, with 95% confidence intervals (CIs) provided. A probability (*p*) value < 0.05 was considered statistically significant.

## 3. Results and Discussion

**Figure 2:**
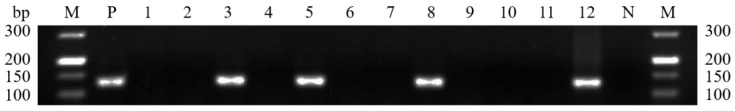
PCR products targeting the *Toxoplasma gondii* B1 gene were amplified from sheep muscle samples, with *T. gondii* DNA derived from tachyzoites of the RH strain as positive control (P). Lanes 1–12 represent the PCR results from DNA extracted from representative sheep muscle samples, lane N represents the negative control, and lane M represents the DNA size markers.

The results of semi-nested PCR showed that 155 out of 755 sheep tissue samples were positive for *T. gondii*, with a molecular prevalence of 20.5%. The prevalence of *T. gondii* infection in sheep varied significantly among the ten areas. Regarding the sampling tissues, the prevalence rates were 21.9% (16/73) in lymphatic tissues and 20.4% (139/682) in muscle tissues, with no statistically significant difference between them (χ^2^ = 0.095, *p* > 0.05). The representative PCR results from sheep muscle samples are shown in Figure 2. The prevalence found in this study was similar to the 17.8% seroprevalence of *T. gondii* in sheep previously reported in Shanxi Province using ELISA assay [17], indicating a consistent level of infection across different studies in the region. Moreover, the prevalence of *T. gondii* infection in different regions of Shanxi Province varied, with the highest prevalence observed in Southern Shanxi (23.0%), followed by Northern Shanxi (20.2%) and Central Shanxi (17.6%), as shown in Table 1, and the geographical location was a risk factor (χ^2^ = 0.095, *p* < 0.05) associated with *T. gondii* infection in sheep in Shanxi Province.

Globally, the prevalence of *T. gondii* in sheep varies significantly across different regions [22]. In this study, the prevalence of *T. gondii* in sheep in Shanxi Province (20.5%) was similar to that reported in Spain (24.5%, molecular prevalence) [23], Egypt (24.0%, seroprevalence) [24], and Algeria (25.6%, seroprevalence) [25]. In contrast, the prevalence of *T. gondii* in sheep in Shanxi Province was lower than that reported in Italy (49.9%, seroprevalence) [26], and the neighboring Mongolia (34.8%, seroprevalence) [27], highlighting regional differences in prevalence. A meta-analysis showed that the overall prevalence of *T. gondii* in sheep in China is 8.5% [28]. Compared with other provinces in China, the prevalence of *T. gondii* in sheep in Shanxi Province (20.5%) was similar to that in Qinghai Province (21.3%, seroprevalence) [29], but higher than that in Shandong Province (9.8%, molecular prevalence) [30], indicating potential regional variations in prevalence. However, due to differences in detection methods, direct cross-regional and cross-method comparisons have limitations: they may overestimate actual active infection rates in regions using serological testing or underestimate long-term infection risks in regions applying molecular detection [31]. Therefore, further studies are required to enhance data consistency. Variation in prevalence across different regions can be attributed to multiple factors, such as the age of the sheep, farming practices, grazing density, sample size and even the breed of sheep. Overall, the prevalence of *T. gondii* in sheep in Shanxi Province remains relatively high.

To further investigate the genetic structure of *T. gondii* in sheep in Shanxi Province, we performed Mn-PCR-RFLP on 155 positive samples to amplify the 12 genetic markers of *T. gondii*. Due to the low DNA concentration of *T. gondii* in most positive samples, only one sample was amplified at all of the 12 genetic markers, one sample was amplified at 11 genetic markers, one sample was amplified at 9 genetic markers, and six samples were amplified at 8 genetic markers. The electrophoresis banding patterns of restriction enzyme digestions are shown in Figure S1. One genotype, ToxoDB#9, was identified in this study (Table 2). Mn-PCR-RFLP remains the primary method for genotyping *T. gondii*, which enhances the resolution and specificity of identification of *T. gondii* genotypes by expanding the number of genetic markers from 9 to 12 [32]. *T. gondii* is genetically diverse, with different dominant genotypes in different countries and geographic regions [33]. Previous studies have identified several genotypes in China, such as ToxoDB#2 and ToxoDB#4 genotypes from lambs in Henan Province [34], and ToxoDB#225 from lambs in Central China [35]. ToxoDB#9 (Chinese 1) is considered to be the dominant genotype of *T. gondii* in China [36], which also has been isolated from sheep in China [37]. In this study, the ToxoDB#9 genotype was detected in one sheep sample in Shanxi Province; however, comprehensive surveillance with larger sample sizes across diverse regions is required to validate its epidemiological predominance. In addition, 11 genetic markers were amplified in one sample and it was suspected to be ToxoDB#52, highlighting the genetic diversity of *T. gondii* in Shanxi Province and the potential for further investigation into the presence of other genotypes in sheep across Shanxi Province.

Molecular identification and genotyping of *T. gondii* in various animals will play a pivotal role in future research on *T. gondii* diversity and control strategies in China. As an established technique for molecular genotyping of *T. gondii*, Mn-PCR-RFLP has been widely utilized in epidemiological surveillance of *T. gondii* globally and in China [21,37]. Amidst the evolution of new technologies, Mn-PCR-RFLP retains its cost-effective utility, particularly by providing sustainable monitoring solutions for toxoplasmosis in resource-limited regions.

In outbreaks of acute human toxoplasmosis documented in Canada and similar regions, the vast majority of cases are linked to consumption of game meat or ingestion of oocysts from environmental sources (e.g., contaminated water), with epidemiological connections to wild felids [38]. Anthropogenic expansion into wildland habitats may facilitate contact between domestic cats, wild felids, and sylvatic intermediate hosts of *T. gondii*, while concurrently increasing human exposure to oocysts shed by wild felids [39]. This heightened overlap among populations potentially elevates risks of human contact with atypical (and potentially more virulent) strains of the parasite [39].

This study revealed the first molecular prevalence of *T. gondii* infection in sheep in Shanxi Province. The results showed that there was *T. gondii* infection in mutton sold for human consumption in the region, underscoring the need for heightened awareness regarding the risks of consuming undercooked mutton. Public health campaigns should emphasize the importance of proper cooking to prevent toxoplasmosis. Additionally, the study highlights the critical importance of controlling *T. gondii* infections in sheep for both human health and socio-economic development. Measures such as minimizing contact between sheep and stray animals, especially cats which can excrete millions of oocysts and can transmit the infection to many hosts, are essential [40]. Furthermore, regular disinfection of farming facilities, along with ensuring access to clean drinking water and feed, is crucial for preventing the spread of toxoplasmosis and maintaining public health and safety.

## 4. Conclusions

This study provides the first comprehensive assessment of the molecular prevalence and genotype of *T. gondii* in sheep in Shanxi Province. The overall molecular prevalence of *T. gondii* in sheep in Shanxi Province was found to be high, at 20.5%. Genotyping revealed the presence of the ToxoDB#9 genotype in sheep in Shanxi Province, which is considered the dominant genotype in China. This study characterized *T. gondii* infection in sheep from Shanxi Province, providing epidemiological data to support toxoplasmosis prevention and control strategies in the region.

## Figures and Tables

**Table 1:** Molecular prevalence of *Toxoplasma gondii* in commercial mutton in different cities of Shanxi Province.

Geographical Location	Category (County)	No. Tested	No. Positive	Prevalence % (95% CI)	*p*-Value
Central Shanxi	Qi	63	6	9.5 (2.3–16.8)	<0.05
	Taigu	64	14	21.9 (11.8–32.0)	
	Pingyao	78	16	20.5 (11.6–29.5)	
Northern Shanxi	Youyu	91	26	28.6 (19.3–37.9)	
	Huairen	95	9	9.5 (3.6–15.4)	
	Hunyuan	72	17	23.6 (13.8–33.4)	
Southern Shanxi	Fushan	60	16	26.7 (15.5–37.9)	
	Jishan	104	23	22.1 (14.1–30.1)	
	Xia	66	16	24.2 (13.9–34.6)	
	Hejin	62	12	19.4 (9.5–29.2)	
Total		755	155	20.5 (17.7–23.4)	

**Table 2:** Genotypic identification of *Toxoplasma gondii* in commercial mutton in Shanxi Province.

Isolate	Host	Location	SAG1	5′ + 3′ SAG2	Alte. SAG2	SAG3	BTUB	GRA6	c22-8	c29-2	L358	PK1	**Apico**	**Genotype**
GT1	Goat	United States	I	I	I	I	I	I	I	I	I	I	I	Reference, Type I, ToxoDB#10
PTG	Sheep	United States	II/III	II	II	II	II	II	II	II	II	II	II	Reference, Type II, ToxoDB#1
CTG	Cat	United States	II/III	III	III	III	III	III	III	III	III	III	III	Reference, Type III, ToxoDB#2
MAS	Human	France	u-1	I	II	III	III	III	u-1	I	I	III	I	Reference, ToxoDB#17
TgCgCa1	Cougar	Canada	I	II	II	III	II	II	II	u-1	I	u-2	I	Reference, ToxoDB#66
TgCatBr5	Cat	Brazil	I	III	III	III	III	III	I	I	I	u-1	I	Reference, ToxoDB#19
TgCatBr64	Cat	Brazil	I	I	u-1	III	III	III	u-1	I	III	III	I	Reference, ToxoDB#111
TgRsCr1	Toucan	Costa Rica	u-1	I	II	III	I	III	u-2	I	I	III	I	Reference, ToxoDB#52
SJS99	Sheep	Yuncheng City	u-1	II	II	III	III	II	II	III	II	II	I	ToxoDB#9
STG50	Sheep	Jinzhong City	II/III	I	II	III	N	III	u-2	II	II	III	I	Suspected, ToxoDB#52

N—no data available. I, II, III—alleles corresponding to the DNA sequences of *T. gondii* clonal lineages I, II, and III. u-1, u-2—alleles distinct from those of lineages I, II, and III.

## Data Availability

The original contributions presented in this study are included in the article and supplementary material. Further inquiries can be directed to the corresponding authors.

## Supplementary Materials

The following supporting information can be downloaded at: https://www.mdpi.com/article/10.3390/ani15121685/s1, Figure S1: PCR-RFLP results of *T. gondii* isolates from sheep at 12 loci.

## Abbreviations

PCR: polymerase chain reaction; Mn-PCR-RFLP: The multilocus nested PCR-restriction fragment length polymorphism.
