# Supplementary material for: Molecular Prevalence and Genotyping of Toxoplasma gondii in Sheep Tissues Intended for Human Consumption in Shanxi Province, North China
Source: Animals (Basel). 2025 Jun 6;15(12):1685. doi: 10.3390/ani15121685 (PMC12189114; doi:10.3390/ani15121685)
Supplement: Supplementary file 1 [file animals-15-01685-s001.zip › animals-3653399-supplementary.pdf]

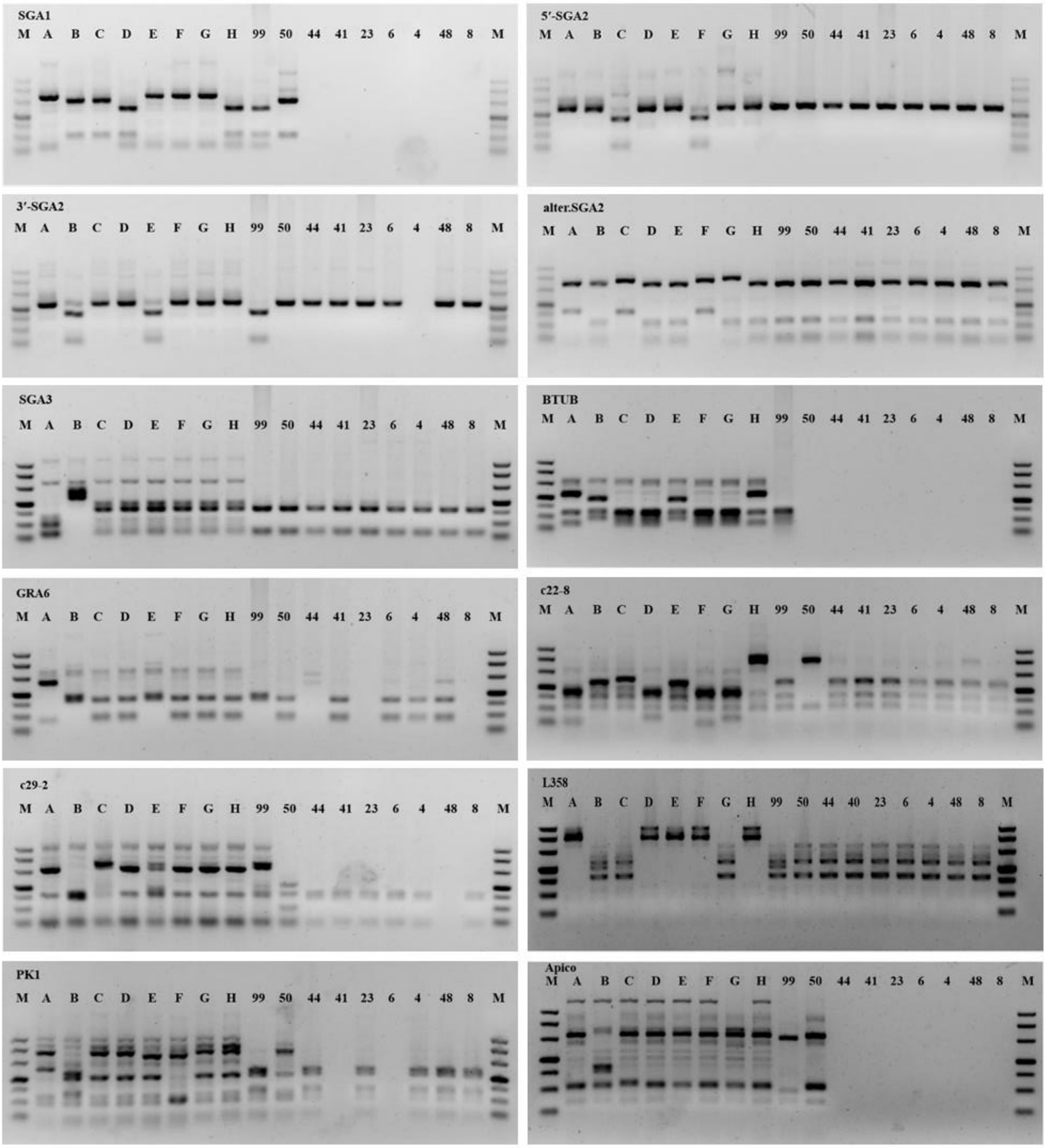

Figure S1: PCR-RFLP results of *Toxoplasma gondii* isolates from sheep at 12 loci. M: DL500 marker; A-H: Reference strains GT1, PTG, CTG, MAS, TgCgCa1, TgCatBr5, TgCatBr64, and TgToucan (TgRsCr1); 99, 50, 44, 41, 23, 6, 4, 48, 8: Positive samples.
